# *In vitro* evaluation of *Rhus succedanea* extracts for ruminants

**DOI:** 10.5713/ajas.18.0045

**Published:** 2018-04-12

**Authors:** Do Hyung Kim, Shin Ja Lee, Da Som Oh, Il Dong Lee, Jun Sik Eom, Ha Young Park, Seong Ho Choi, Sung Sill Lee

**Affiliations:** 1 Department of Animal Science, Gyeongbuk Provincial College, Yecheon 36830, Korea; 2 Institute of Agriculture and Life Science and University-Centered Labs, Gyeongsang National University, Jinju 52828, Korea; 3 Division of Applied Life Science (BK21 Program) and Institute of Agriculture & Life Science (IALS), Gyeongsang National University, Jinju 52828, Korea; 4 Department of Pathology, Busan Paik Hospital, Inje University College of Medicine, Busan 47392, Korea; 5 Department of Animal Science, Chungbuk National University, Cheongju 28644, Korea

**Keywords:** *Rhus succedanea* Extracts, *In vitro* Ruminal Fermentation, Microbial Growth

## Abstract

**Objective:**

This study was conducted to evaluate the effects of *Rhus succedanea* extract addition on *in vitro* ruminal fermentation and microbial growth.

**Methods:**

Two ruminally-fistulated steers consuming 600 g/kg timothy- and 400 g/kg cracked corn-based concentrate with free access to water and mineral block were used as rumen fluid donors. *In vitro* batch fermentation, with timothy as a substrate, was conducted for up to 72 h, with *Rhus succedanea* extracts added to achieve final concentrations of 0, 10, 30, 50, 70, and 90 mg/L.

**Results:**

Effective dry matter (DM) degradability rate linearly decreased (p = 0.046) depending on extract dosing levels. Total gas production after 24 to 72 h incubation tended to decrease following extract addition, beginning with 50 mg/L starting dose (significance of quadratic effects: p = 0.006, p<0.001, and p = 0.008 for 24, 48, and 72 h, respectively). Methane production decreased depending on dosing levels following 24 h (p<0.05) and 48 h (p<0.005) incubations and was the lowest with the 50 mg/L dose. The *Rhus succedanea* extracts increased the abundance of *Fibrobacter succinogenes* (p<0.05) and *Ruminococcus flavefaciens* (p = 0.0597) and decreased the abundance of methanogenic archaea (p<0.05) following 24 h incubation.

**Conclusion:**

*Rhus succedanea* was shown to reduce methane production and increase cellulolytic bacteria without any signs of toxic effects and with a minor effect on DM degradability.

## INTRODUCTION

The efficiency of ruminal fermentation can be facilitated by modifying the feeding regime of ruminants using natural feed additives. A number of methanogenic inhibitors have been developed to improve feed conversion efficiency of ruminant feeds, which are claimed to be effective in suppressing methanogens or overall bacterial activities [1]. However, some compounds are toxic or may not be economically feasible, or an adaptive response may occur in some bioactive compounds after supplementation.

In recent years, there has been a global trend toward the use of natural plants as medicinal and functional food additives. Medicinal plants have various characteristics, such as antimicrobial, antiviral, and immune system stimulating activities, which can be beneficial to animal health and production. *Rhus succedanea*, the wax tree, is a flowering plant species found in Asia. *Rhus succedanea* (formerly *Toxicodendron succedaneum*) has been used in indigenous medicine for quite a long time in the treatment of asthma, cough, and colicky pains [2], and has anti-rumor, anti-oxidation, hangover cure, and gastritis suppression effects [3]. Bioactive constituents from *R. succedanea* have been isolated and characterized. These mostly include urushiol, flavonoids, and phenols [3].

Although a number of studies using plant extracts have been conducted to apply them as feed additives, *R. succedanea* extracts have rarely been applied to ruminant animals. Kim et al [4] reported that dietary *Rhus verniciflua* supplementation of Hanwoo cattle feed was effective in increasing meat color stability, water-holding capacity, and unsaturated fatty acid content, as well as retarding lipid oxidation. However, information on the effects of application of *R. succedanea* on ruminal fermentation is limited. Therefore, this study was conducted to evaluate the effects of *R. succedanea* extracts on *in vitro* ruminal metabolites, gas production, and microbial growth.

## MATERIALS AND METHODS

### Sample preparation

*Rhus succedanea* extracts were obtained from the Plant Extract Bank (KRIBB, Daejeon, Korea). *R. succedanea* was cut into small pieces and dried naturally under shade. Extraction from the dried pieces (100 g each) was then performed with 99.9% methyl alcohol (1,000 mL) using an ultrasonic cleaner (Branson Ultrasonics Corporation, Danbury, CT, USA) at room temperature for three days. After extraction, the solutions were filtered and the solvents were evaporated under vacuum conditions. Stock solution (20 mg/mL) of the extracts was dissolved in dimethyl sulfoxide (Sigma-Aldrich Chemical Co., St. Louis, MO, USA) and diluted using culture medium immediately before *in vitro* incubation. *R. succedanea* extracts were prepared to achieve final dosing concentrations of 0, 10, 30, 50, 70, and 90 mg/L.

### *In vitro* batch fermentation

Ruminal contents were collected from two ruminally-fistulated steers (with mean body weight±standard error of 450±30 kg), which had been consuming 600 g/kg body weight timothy and 400 g/kg cracked corn-based concentrate (crude protein, 120 g/kg; ether extracts, 15 g/kg; crude fiber, 150 g/kg; crude ash, 120 g/kg; Ca, 7.5 g/kg; P, 9.0 g/kg; total digestible nutrients, 690 g/kg dry matter [DM] basis) with free access to water and mineral block (supplied per kilogram of diet: vitamin [Vit] A, 3,800 IU; Vit E, 400 IU; Vit E, 500 IU; Fe, 7 mg; Cu, 2.4 mg; Mn, 30 mg; Zn, 6.0 mg; I, 1.5 mg; Se and Co, 1.5 mg). The diet was fed at 3% of body weight of the steers in two equal portions at 07h00 and 17h00 daily. The steers were acclimated to the diet for a minimum of 14 days.

On the day of fermentation testing, approximately 2 kg of ruminal contents were collected from the dorsal, ventral, and caudal rumen of each steer 2 h after the morning feeding, and collected into an insulated container for transport to the laboratory, usually within 10 min after collection. Ruminal contents were processed with a Waring blender under a CO_2_ atmosphere and strained through four layers of cheesecloth and glass wool prior to combining with McDougall buffer [5]. The McDougall buffer (1,000 mL) and ruminal inoculums (500 mL) were combined, and 25 mL of this mixture was then added to 60-mL fermentation vessels (serum bottles) containing 0 or 300 mg (based on DM) of substrate. The substrate was supplied from the timothy that was fed to the steers, and was used after being ground up in a Wiley mill until it could pass through a 2 mm screen. The fermentation vessels were closed with butyl rubber stoppers under the anaerobic gassing systems while being connected to a source of oxygen-free gas, and then were sealed with aluminum caps and placed in an incubator at 39°C for 3, 6, 9, 12, 24, 48, and 72 h without shaking. Fermentation was conducted in a completely randomized order and in duplicate for each sample, and was then replicated on three separate days (n = 3 for each dose in statistical analyses).

### Total gas and methane production, ammonia-nitrogen, and volatile fatty acid content

At the end of each incubation, a detachable pressure transducer and a digital readout voltmeter (Laurel Electronics, Inc., Costa Mesa, CA, USA) were used to measure the headspace gas pressure in each vessel. Gas samples for methane analysis were drawn from each vessel into sampling syringes and transferred into a vacuum test tube (Vacutainer, Becton Dickinson, Franklin Lakes, NJ, USA). Gas samples were analyzed for methane concentrations by gas chromatography (Agilent Technologies HP 5890, Santa Clara, CA, USA) conducted using a thermal conductivity detector with a Column Carboxen 1006PLOT capillary column, measuring 30 m×0.53 mm (Supelco, Bellefonte, PA, USA), as described by Zafarian and Manafi [6].

After determination of gas production, the vessels were uncapped, the pH of fermentation media was then measured, and a 5-mL aliquot of the fermentation medium was combined with 0.5 mL of 2-ethylbutyrate (85 mM) as an internal standard and 0.5 mL of 50 g/kg metaphosphoric acid for analysis of volatile fatty acid (VFA) concentrations. These samples were centrifuged at 39,000×g at 23°C for 15 min, transferred to vials, capped, and analyzed for VFA concentrations by gas chromatography (model GC-14B, Shimadzu Co. Ltd., Tokyo, Japan) using a Thermon-3000 5% Shincarbon A columm (1.6 m×3.2 mm i.d., 60 to 80 mesh, Shinwakako, Kyoto, Japan) and flame-ionization detector (column temperature = 130°C, injector and detector temperature = 200°C). The carrier gas (N_2_) flow rate was 50 mL/min. A 5-mL aliquot of the fermentation medium was combined with 0.5 mL of 25 g/kg metaphosphoric acid for analysis of NH_3_-N concentration using a spectrophotometer (Model 680, Bio-Rad Laboratories, Hercules, CA, USA) and methods based on glutamate dehydrogenase.

### Relative quantification of specific ruminal microbes

Total nucleic acid was extracted from the incubated rumen samples using the modified bead-beating protocol with the Soil kit (Macherey-nagel, Düren, Germany). This was accomplished by taking a 1.0-mL aliquot from the culture medium using a wide-bore pipette to ensure collection of a homogeneous sample, and then centrifugating the aliquot at 39,000×g. Nucleic acid concentrations were measured using a NanoDrop Spectrophotometer (Thermo Scientific, Wilmington, DE, USA).

Polymerase chain reaction (PCR) primer sets were then used in this study to detect and amplify DNA from *Fibrobacter succinogenes* (forward primer: GTT CGG AAT TAC TGG GCG TAA A; reverse primer: CGC CTG CCC CTG AAC TAT C), *Ruminococcus flavefaciens* (forward primer: CGA ACG GAG ATA ATT TGA GTT TAC TTA GG, reverse primer: CGG TCT CTG TAT GTT ATG AGG TAT TAC C), and *Ruminococcus albus* (forward primer: CCC TAA AAG CAG TCT TAG TTC G; reverse primer: CCT CCT TGC GGT TAG AAC A), and the primers used were the same as those referenced by Denman and McSweeney [7], Koike and Kobayashi [8], and Skillman et al [9], respectively. A total bacteria primer set (forward: CGG CAA CGA GCG CAA CCC; reverse: CCA TTG TAG CAC GTG TGT AGC C) was used as the internal standard [7].

Real-time PCR (RT-PCR) assays for enumeration of microbes were performed according to the methods described by Denman and McSweeney [7] and Denman et al [10] on a real-time PCR Machine (CFX96 Real-Time system, Bio-Rad, USA) using the SYBR Green Supermix (QPK-201, Toyobo Co., Ltd., Tokyo, Japan). The values of the cycle threshold (Ct) after PCR reactions were used to determine fold change (number of fold difference) of different microbial populations relative to the control without additives. Abundance of these microbes was expressed by the equation: relative quantification = 2^−ΔCt(Target) −ΔCt(Control)^, where Ct represents the threshold cycle. All RT-PCR reaction mixtures (final volume of 25 μL) contained forward and reverse primers (10 pmol each), the SYBR Green Supermix (QPK-201, Toyobo Co., Ltd., Japan), and DNA templates ranging from 10 to 100 ng. A negative control without template DNA was used in every RT-PCR assay for each primer. The amplification of the target DNA was performed as described by Denman and McSweeney [7], Koike and Kobayashi [8], and Skillman et al [9].

### Calculations and statistical analysis

To give a more precise estimate of gas production throughout fermentation, the following calculation was used to analyze the kinetic data, as described by Ørskov and McDonald [11]: G_P_ = a+b(1–exp^−c×time^), where G_P_ is gas production (mL/g DM of substrate) at time t; a, b, and c are the scaling factors for the Y-axis intercept (mL/g of DM), potential gas production (mL/g of DM), and the rate constant for gas production per h, respectively. Gas production rate was fitted to the model by using the NLIN procedure of SAS (version 9.1, SAS Inst. Inc., Cary, NC, USA) employing Marquadt’s algorithm, while varying a, b, and c. Effective gas production (EG_P_, i.e. substrate availability) from the culture was estimated as EG_P_ = a+b(k_d_/[k_d_+k_p_]), where k_d_ is a gas production rate constant, and k_p_ is a passage rate constant assumed to be 0.05/h [12].

Data obtained from the experiment were analyzed using the general linear model procedure of SAS (SAS Inst. Inc., USA) for a completely random design. The model included terms for dosing levels, time, and their interaction. Orthogonal contrast was used to assess linear, quadratic, and cubic relationships between the dosing levels of *R. succedanea* extracts and the dependent variables. Orthogonal coefficients for unequally spaced dosing were acquired using the IML procedure (SAS Inst. Inc., USA).

## RESULTS

Table 1 shows the effects of different doses of *R. succedanea* extracts on DM degradability and their parameters after different incubation periods. The DM degradability was not affected by the dose of *R. succedanea* extracts, except after 24 and 72 h incubations, when it decreased by dose (significance of linear effect: p = 0.04; linear and cubic effects: p = 0.014 and p = 0.041, respectively). The DM degradability for 24 and 72 h incubations was decrease by 70 and 90 mg/L doses of *R. succedanea* extracts. Effective DM degradability rate (E_DM_) decreased linearly with dose (p = 0.046). Cumulative gas production rapidly increased from 12 to 72 h of incubation (Table 2). The *R. succedanea* extracts increased total gas production depending on dosing level at 24 (linear, quadratic, and cubic effects: p = 0.005, 0.006, and 0.051, respectively), 48 (quadratic effect: p<0.001), and 72 h (quadratic and cubic effects: p = 0.008 and 0.031, respectively) incubations. In addition, the total gas production for 24 to 72 h incubation tended to decrease with 50 mg/L dosing of *R. succedanea* extract as a starting point (quadratic effects: p = 0.006, p<0.001, and p = 0.008 for 24, 48, and 72 h, respectively). The potential gas production (a+b) was decreased (cubic effect: p = 0.051) by dosing with 50 mg/L of *R. succedanea* extracts. The decrease in the potential gas production led to an increase in the k value. Effective gas production rate (E_Gp_) was decreased (linear and quadratic effects: p = 0.003 and 0.069, respectively) depending on dosing levels.

**Figure 1:**
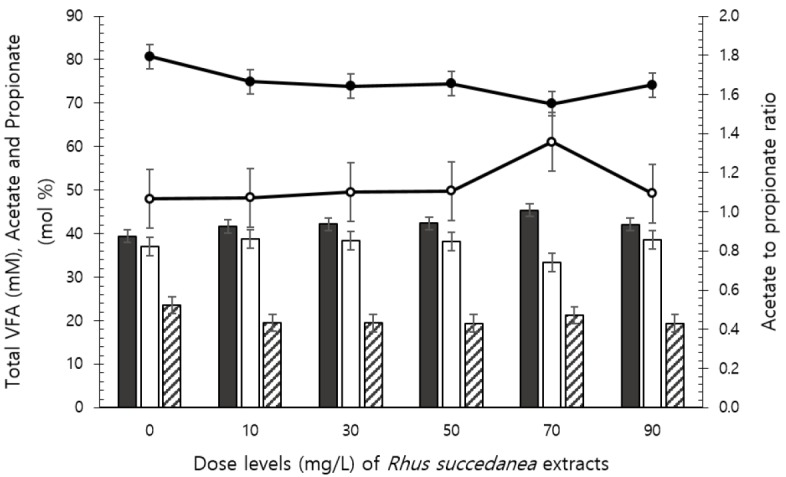
*In vitro* ruminal fermentation characteristics at 24 h incubation by different doses of *Rhus succedanea* extracts. Total volatile fatty acid (filled circle), acetate to propionate ratio (open circle), acetate (solid bar), propionate (open bar), and butyrate (hatched bar) concentrations. Error bars are standard error of the mean.

The pH was slightly increased after fermentation depending on the dose used at 24 h and 72 h, but remained within an optimal pH range of 6 to 7 (Table 3). Ammonia-N concentration changed in a quadratic manner (p = 0.003) following 24 h incubation, which was increased by 50 mg/L of *R. succedanea* extracts. Total VFA concentration was decreased by 50 mg/L of *R. succedanea* extracts following 48 h (quadratic effect: p = 0.021) and by 70 and 90 mg/L of *R. succedanea* extracts following 72 h incubation (linear effect: p = 0.014). Total VFA concentration following 24 h of incubation decreased (p<0.05) and acetate concentration increased (p<0.05) relative to control with dosing of 70 mg/L of *R. succedanea* extracts (Figure 1). No differences were observed in propionate and butyrate concentrations and acetate to propionate ratio. Methane production was decreased depending on dosing levels following 24 h (linear and quadratic effects: p<0.0001 and p = 0.002, respectively) and 48 h (linear and cubic effects: p<0.0001 and p<0.001, respectively; Table 4) incubations, and was the lowest at 50 mg/L doses of *R. succedanea* extract.

**Figure 2:**
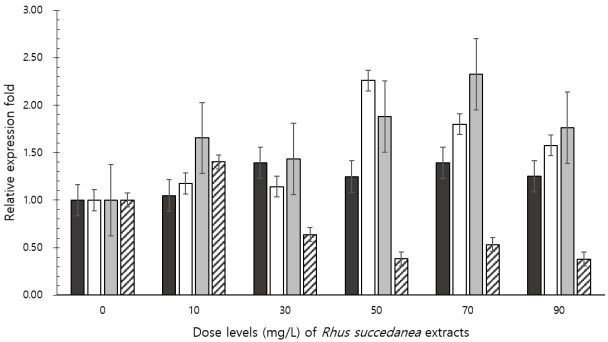
Relative quantification of *in vitro* rumen microbial populations at 24 h incubation by different doses of *Rhus succedanea* extracts. *Ruminococcus albus* (black bar), *Fibrobacter succinogenes* (white bar), *Ruminococcus flavefaciens* (grey bar), and methanogenic archaea (hatched bar). Error bars are standard error of the mean.

Real-time PCR analyses indicated that *R. succedanea* extracts affected the abundance of cellulolytic bacteria and methanogenic archaea (Figure 2). The dose of *R. succedanea* extracts increased the abundance of *Fibrobacter succinogenes* (linear, quadratic, and cubic effects: p<0.0001, p = 0.0006, and p = 0.0078, respectively) and *Ruminococcus flavefaciens* (linear effect: p = 0.0597), and decreased the abundance of methanogenic archaea (linear and quadratic effects: p<0.0001 and p = 0.0073, respectively) after 24 h of incubation.

## DISCUSSION

Total gas production was closely related to the digestion of fermentation substrates, VFA production, and microbial activity and growth [13]. Ruminal microbial activity was affected by the use of plant extracts and secondary plant metabolites [14]. In the present study, EGP decreased in response to the increasing doses of *Rhus succedanea* extracts. Similarly, E_DM_ linearly decreased with increasing *R. succedanea* extract doses. However, the rate of the decrease in E_GP_ in response to the dose of *R. succedanea* extracts tended to slow at 50 mg/L dosing of *R. succedanea* extracts (quadratic effect: p = 0.069). Similarly, total VFA concentration was decreased following 48 and 72 h incubations (quadratic effect: p = 0.021; linear effect: p = 0.014). Busquet et al [14] reported that at the highest concentrations of various plant extracts, most treatments showed decreased total VFA production, possibly reflecting decreased feed digestion. Plant secondary metabolites are particularly attractive as rumen modifiers that are generally accepted to be environmental friendly and safe to use in food production systems. Due to their potential to adversely affect feed intake and nutrient utilization, however, these should be administered at low concentrations to beneficially alter ruminal fermentation. The effect of *R. succedanea* extract dose on ammonia-N concentration was quadratic (p = 0.003) following 24 h and 48 h incubations, and this effect was increased for 50 mg/L extracts and decreased for 70 mg/L extracts. These results suggest that changes observed to be caused by *R. succedanea* extract on ruminal ammonia-N concentration may be contradictory depending on the dose used. The observed reduction in ammonia-N suggests that over 70 mg/L of *R. succedanea* extracts reduced amino acid deamination. Inhibition of amino acid deamination has practical implications because it may increase ruminal use of dietary protein and improve the efficiency of N use in the rumen [15].

Ruminal cellulolysis is conducted primarily by *Fibrobacter succinogenes*, *Ruminococcus flavefaciens*, and *Ruminococcus albus* [16], and their relative populations can potentially impact the ratios of VFA available to ruminants. In the present study, an increase in ruminal acetate concentration was observed to accompany the addition of 70 mg/L dose of *R. succedanea* extracts, and this was consistent with the observed increase in abundance of the gram-negative bacteria *Fibrobacter succinogenes*. This bacterium intensively degrades plant cell walls by an erosion-like mechanism, in which it burrows its way through the complex matrix of cellulose and hemicellulose in the cell wall resulting in the release of digestible and undigested cell wall fragments [17]. *Ruminococcus flavefaciens*, a gram-positive bacteria, was also observed to increase with addition of 70 mg/L dose of *R. succedanea* extract that corresponded with the increase of acetate concentration. Production of methane and propionate are negatively correlated because both these processes compete for hydrogen [18]. However, the negative relationship between propionate concentration and methane output was not evident from our results. Formation of acetate and butyrate results in production of additional methanogenic substrates, which are formate and hydrogen, and propionate formation results in less hydrogen being available for methane production [18]. In addition, low methane production might be related to reduced fiber digestibility, thus, also influencing the energy input to the animal. In the present study DM degradability and gas production linearly decreased, whereas *Fibrobacter succinogenes* and *Ruminococcus flavefaciens*, which are considered to be primarily responsible for plant cell wall biodegradation, increased after 24 h of incubation. Nevertheless, the decreasing methane production and abundance of methanogenic archaea observed with extract dosing were likely caused by the combined effects of decreased total VFA concentration and decreased DM fermentation. *R. succedanea* produces a cytotoxic biflavonoid, and it has been reported that the methanol extract of *Rhus succedanea* showed positive indications of the presence of phenols, steroids, alkaloids, flavonoids, tannins, glycosides, and carbohydrates [19]. It has been documented that alkaloids, flavonoids, tannins, and phenols are plant secondary metabolites, which are well-known for their antimicrobial activity in the rumen. The extract of stem bark of *Rhus* had antioxidant effects against hydroxyl radicals and antiproliferative activity against human cancer cell lines, and also augmented the activity of cell-associated detoxifying enzymes in hepatocytes [20–22]. The sap of the wax tree (*R. succedanea*) is composed of urushiol, glycoprotein, flavonoids, a gummy substance that contains laccase, stellacyanin, polysaccharides, peroxidase, and water [23]. Therefore, the observed antibacterial potency of methanol extracts from *R. succedanea* can be attributed to the nature of its biologically active components, which might be enhanced in the rumen. However, further work is needed to clarify the relationship between fibrolytic microbes and methanogens, and although suppression of methanogenic archaea by *R. succedanea* extracts was observed in this study, it must be noted that the long-term effects of the extracts might be different because adaptation of the rumen microbes might occur.

## Figures and Tables

**Table 1:** Effects of different doses of *Rhus succedanea* extracts on *in vitro* dry matter degradability by mixed rumen anaerobic microbial fermentation

Incubation time	Dose (mg/L)						SEM	Contrast		
	
	0	10	30	50	70	90		Linear	Quadratic	Cubic
Dry matter degradability (%)										
3 h	18.84	19.32	18.75	17.98	18.41	18.15	0.25	0.006	0.443	0.314
6 h	19.44	18.89	18.69	19.82	19.20	19.31	0.37	0.586	0.889	0.182
9 h	20.42	20.26	20.11	19.01	19.60	20.14	0.50	0.326	0.126	0.426
12 h	18.79	19.04	18.33	18.79	18.46	18.02	0.68	0.370	0.827	0.748
24 h	23.01	23.57	21.28	23.55	21.04	21.94	0.56	0.040	0.648	0.977
48 h	34.91	35.68	34.12	30.95	35.12	34.54	1.15	0.514	0.086	0.815
72 h	42.13	39.89	39.36	40.05	39.66	38.97	0.61	0.014	0.250	0.041
Dry matter degradability parameters1)										
a (%)	8.87	8.08	8.67	7.49	8.71	8.23	0.58	0.673	0.512	0.686
b (%)	35.20	31.80	32.47	31.53	33.17	31.57	1.52	0.317	0.485	0.236
a+b (%)	44.07	39.88	41.15	39.02	41.89	39.77	2.07	0.388	0.491	0.319
k (D_M_/h)	0.0325	0.0425	0.0347	0.0527	0.0325	0.0390	0.01	0.856	0.496	0.755
E_DM_	22.49	22.69	21.66	21.90	21.72	21.85	0.31	0.046	0.181	0.942

SEM, standard error of the mean, n = 3.

1) Potential extent and rate of dry matter degradability were determined using the exponential model: D_M_ = a+b(1–exp^−c×time^), where D_M_ is dry matter degradability (%) at time t; a = dry matter degradability from the immediately soluble fraction; b = dry matter degradability from the insoluble fraction; c = the fractional rate of dry matter degradability per hour; a+b = potential extent of dry matter degradability; E_DM_ = effective dry matter degradability rate from the cultures, calculated as E_DM_ = a+b[k_d_/(k_d_+k_p_)], where k_d_ is a dry matter degradability rate constant, and k_p_ is a passage rate constant assumed to be 0.05 h^−1^.

**Table 2:** Effects of different doses of *Rhus succedanea* extracts on *in vitro* cumulative gas production by mixed rumen anaerobic microbial fermentation

Incubation time	Dose (mg/L)						SEM	Contrast		
	
	0	10	30	50	70	90		Linear	Quadratic	Cubic
Gas production (mL/0.1 g DM of substrate)										
3 h	14.31	14.41	14.43	14.31	14.23	14.21	0.08	0.085	0.285	0.228
6 h	15.30	15.40	15.26	15.16	15.35	15.17	0.11	0.315	0.885	0.746
9 h	16.03	16.28	15.47	15.83	15.00	16.08	0.22	0.100	0.023	0.058
12 h	17.29	16.77	16.85	16.74	16.63	16.18	0.21	0.006	0.744	0.202
24 h	19.08	19.32	19.34	19.33	18.90	18.86	0.09	0.005	0.006	0.051
48 h	24.99	25.31	24.85	21.13	25.64	25.64	0.42	0.786	<0.001	0.240
72 h	31.34	32.14	32.70	32.34	31.72	31.72	0.28	0.872	0.008	0.031
Gas production parameters1)										
a (mL/0.1 g DM of substrate)	4.80	5.41	5.85	4.47	5.71	5.67	0.30	0.182	0.782	0.096
b (mL/0.1 g DM of substrate)	22.83	23.47	23.95	21.74	23.77	23.64	0.30	0.345	0.207	0.046
a+b (mL/0.1 g DM of substrate)	27.63	28.87	29.80	26.21	29.47	29.30	0.56	0.221	0.400	0.051
k (G_p_/h)	0.0721	0.0608	0.0517	0.0838	0.0524	0.0541	0.01	0.212	0.530	0.170
E_Gp_	18.24	18.27	18.02	17.84	17.85	17.92	0.98	0.003	0.069	0.408

SEM, standard error of the mean, n = 3; DM, dry matter.

1) Potential extent and rate of gas production were determined using the exponential model: G_P_ = a+b(1–exp^−c×time^), where G_P_ is gas production (mL/g DM of substrate) at time t; a = gas production from the immediately soluble fraction; b = gas production from the insoluble fraction; c = the fractional rate of gas production per hour; a+b = potential extent of gas production; k = gas production rate constant for the insoluble fraction; E_GP_ = effective gas production rate from the cultures, calculated as E_GP_ = a+b[k_d_/(k_d_+k_p_)], where k_d_ is a gas production rate constant, and kp is a passage rate constant assumed to be 0.05 h^−1^.

**Table 3:** Effects of different doses of *Rhus succedanea* extracts on *in vitro* pH, and ammonia-N and total volatile fatty acid concentrations by mixed rumen anaerobic microbial fermentation

Incubation time	Dose (mg/L)						SEM	Contrast		
	
	0	10	30	50	70	90		Linear	Quadratic	Cubic
pH										
3 h	7.04	7.02	7.02	7.01	7.01	6.99	0.02	0.108	0.874	0.556
6 h	6.82	6.83	6.83	6.83	6.83	6.83	0.01	0.132	0.409	0.794
9 h	6.80	6.81	6.80	6.80	6.81	6.80	0.01	0.781	0.700	0.664
12 h	6.81	6.87	6.83	6.83	6.86	6.84	0.02	0.651	0.731	0.860
24 h	6.70	6.73	6.73	6.73	6.75	6.76	0.01	0.001	0.608	0.409
48 h	6.53	6.56	6.57	6.58	6.54	6.53	0.02	0.498	0.035	0.360
72 h	6.37	6.42	6.44	6.43	6.45	6.47	0.01	<0.0001	0.019	0.004
Ammonia-N concentration (mL/dL)										
3 h	2.31	1.76	2.02	2.07	0.47	0.89	0.65	0.067	0.708	0.654
6 h	1.33	2.36	0.22	0.36	0.67	0.69	0.53	0.084	0.155	0.837
9 h	0.53	0.49	0.80	3.42	0.47	0.96	0.61	0.411	0.052	0.769
12 h	0.18	2.96	0.53	0.11	0.40	1.18	0.75	0.528	0.435	0.109
24 h	0.38	0.27	0.44	2.98	0.44	0.40	0.36	0.353	0.003	0.219
48 h	0.56	0.76	0.44	1.16	0.18	0.40	1.53	0.164	0.019	0.290
72 h	2.22	2.11	1.13	1.71	1.73	1.67	0.27	0.203	0.101	0.175
Total VFA concentration (mM)										
3 h	80.60	74.63	75.10	75.95	75.36	76.39	2.15	0.428	0.213	0.310
6 h	75.40	74.20	77.20	75.17	76.77	75.25	1.06	0.580	0.336	0.779
9 h	76.54	74.49	74.67	74.93	76.55	74.69	1.59	0.902	0.797	0.243
12 h	77.43	65.09	73.80	73.10	72.59	76.80	4.93	0.562	0.517	0.627
24 h	80.68	74.96	73.89	74.48	69.81	74.19	2.81	0.084	0.187	0.975
48 h	89.28	88.42	86.24	82.32	87.47	86.80	1.39	0.149	0.021	0.774
72 h	98.87	97.79	96.08	96.57	95.44	95.00	1.01	0.014	0.277	0.266

SEM, standard error of the mean, n = 3; VFA, volatile fatty acid.

**Table 4:** Effects of different doses of *Rhus succedanea* extracts on *in vitro* cumulative methane production by mixed rumen anaerobic microbial fermentation

Incubation time (h)	Dose (mg/L)						SEM	Contrast		
	
	0	10	30	50	70	90		Linear	Quadratic	Cubic
Methane production (mL/g DM of substrate)										
3 h	0.29	0.25	0.24	0.25	0.23	0.23	0.03	0.211	0.591	0.631
6 h	0.68	0.52	0.61	0.57	0.64	0.68	0.05	0.390	0.131	0.385
9 h	1.02	1.05	0.95	0.85	0.79	0.91	0.05	0.004	0.058	0.064
12 h	1.27	0.73	1.07	1.06	1.08	1.10	0.09	0.596	0.341	0.060
24 h	2.19	2.18	1.58	1.59	1.51	1.51	0.08	<0.0001	0.002	0.598
48 h	6.26	7.92	6.83	4.28	4.99	4.77	0.26	<0.0001	0.291	<0.001
72 h	10.49	12.42	10.29	11.92	10.93	10.66	0.44	0.458	0.298	0.852

SEM, standard error of the mean, n = 3; DM, dry matter.
